# A novel therapeutic strategy of lipidated promiscuous peptide against *Mycobacterium tuberculosis* by eliciting Th1 and Th17 immunity of host

**DOI:** 10.1038/srep23917

**Published:** 2016-04-07

**Authors:** Pradeep K Rai, Sathi Babu Chodisetti, Sajid Nadeem, Sudeep K Maurya, Uthaman Gowthaman, Weiguang Zeng, Ashok K Janmeja, David C Jackson, Javed N Agrewala

**Affiliations:** 1 CSIR-Institute of Microbial Technology, Chandigarh, India; 2 Department of Microbiology and Immunology, Peter Doherty Institute for Infection and Immunity, The University of Melbourne, Parkville 3010, Victoria, Australia; 3 Department of Pulmonary Medicine, Government Medical College and Hospital, Chandigarh, India

## Abstract

Regardless of the fact that potent drug-regimen is currently available, tuberculosis continues to kill 1.5 million people annually. Tuberculosis patients are not only inflicted by the trauma of disease but they also suffer from the harmful side-effects, immune suppression and drug resistance instigated by prolonged therapy. It is an exigency to introduce radical changes in the existing drug-regime and discover safer and better therapeutic measures. Hence, we designed a novel therapeutic strategy by reinforcing the efficacy of drugs to kill *Mtb* by concurrently boosting host immunity by L91. L91 is chimera of promiscuous epitope of Acr1 antigen of *Mtb* and TLR-2 agonist Pam2Cys. The adjunct therapy using drugs and L91 (D-L91) significantly declined the bacterial load in *Mtb* infected animals. The mechanism involved was through enhancement of IFN-γ^+^TNF-α^+^ polyfunctional Th1 cells and IL-17A^+^IFN-γ^+^ Th17 cells, enduring memory CD4 T cells and downregulation of PD-1. The down-regulation of PD-1 prevents CD4 T cells from undergoing exhaustion and improves their function against *Mtb*. Importantly, the immune response observed in animals could be replicated using T cells of tuberculosis patients on drug therapy. In future, D-L91 therapy can invigorate drugs potency to treat tuberculosis patients and reduce the dose and duration of drug-regime.

Tuberculosis (TB) is one of the leading global threats accounting for maximum number of deaths caused by a single pathogen^1^. Despite the availability of effective drugs, TB continues to kill approximately 2 million people annually^2^. The problem is further compounded by the fact that treatment of TB patients with drugs leads to the emergence of multi-drug resistant (MDR), extensively drug resistant (XDR) and totally drug resistant (TDR) strains of *Mycobacterium tuberculosis* (*Mtb*)^3^. Globally 20.5% of MDR cases were reported in 2013; out of which 9% were XDR^4^. Unfortunately, cure rate of MDR treatment is less than 50% because of the lack of effective drug regimen^5^. In addition, patients suffer from side-effects of drugs like hepatic and renal toxicity^6^. Adjunct therapy using drugs and vaccines with therapeutic ability can be prudent measure to treat any disease. Since, one third of the world population is latently infected with *Mtb* (LTBI), hence there are fair chances of reactivation of disease when immunity is suppressed^7^. Consequently, development of novel and effective therapeutic strategies to treat TB are urgently required. Long-term prophylactic efficacy of currently available BCG vaccine has been shown in native Alaska and American Indian population in a 60 years of follow-up study^8^. However, it has less scope of employing BCG in a combinatorial therapy with drugs against *Mtb* due to its sensitivity to anti-TB drugs^1^. In the initial step of development of TB vaccine for latently infected population of TB endemic regions, understanding the type of protective immune response is important. Vaccines based on early secretory antigens like MVA85A failed to confer significant protection against TB in randomized clinical trial in endemic regions^9^,^10^. These results suggest that vaccines based on latency associated antigens may be a promising approach against latently infected TB individuals. Recently, we have developed a self-adjuvanting synthetic vaccine construct (L91), which comprises of promiscuous CD4 epitope and TLR-2 agonist-Pam2Cys^11^. L91 exhibits significantly higher protective potential than BCG, as evidenced by decrease in *Mtb* load in the lungs of mice and Guinea pigs^11^. Hence, we thought that it would be wise to examine the therapeutic efficacy of L91 by combinatorial therapy involving drugs and L91. Interestingly, adjunct therapy of L91 with drugs (D-L91), substantially bolstered the effector and memory immunity of the host and decreased the mycobacterial burden in the lungs of *Mtb* infected animals. These results could be replicated using PMBCs of the TB-patients on drug therapy. Therefore, the current approach offers a new insight for the effective treatment of TB patients with D-L91.

## Results

### L91 treatment rescues CD4 T cell from exhaustion and elicits robust Th1 and Th17 immune response

**Figure 1:**
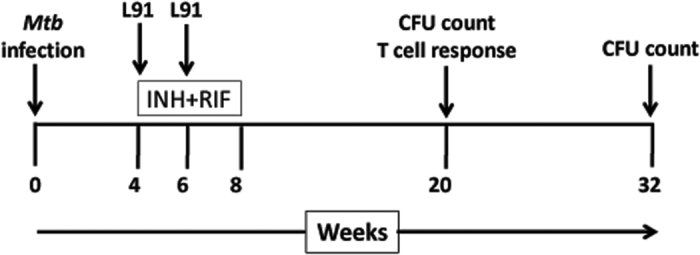
Infection and vaccination schedule. The mice were aerosol challenged with the *Mtb* H37Rv strain and animals were sc immunized twice with L91 at 4 wk and 6 wk of *Mtb* post-infection (pi). The drugs (INH + RIF) were orally administered in drinking water from 4 wk to 8 wk of pi. The bacterial burden in the lungs and spleen was enumerated at 20 wk and 32 wk of pi. *In vitro* T cell recall response was evaluated at 20 wk of pi.

**Figure 2:**
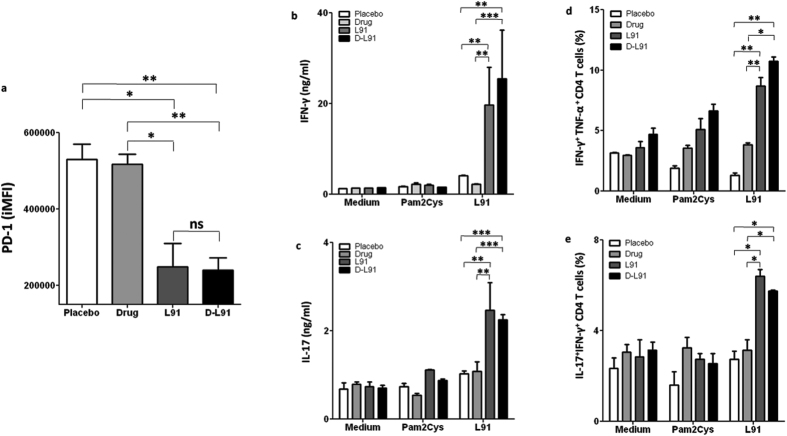
L91 immunization rescues CD4 T cells from exhaustion and activates polyfunctional Th1 cells in the drug treated and *Mtb* exposed mice. At 20 wk of *Mtb* infection, lung cells from D-L91 treated mice were isolated and expression of PD-1 was examined by flowcytometery. (**a**) Bar diagram display integrated mean fluorescence intensity (iMFI) of PD-1 marker on CD4^+^ T cells. Lung cells were isolated from infected mice undergone combinatorial therapy of drug and L91 and single cell suspensions were prepared and *in vitro* stimulated with L91. Control cultures were incubated with Pam2Cys and medium. After 72 h, SNs of stimulated culture were examined for the secretion of (**b**) IFN-γ and (**c**) IL-17A by ELISA. The co-expression of IFN-γ/TNF-α and IL-17A/IFN-γ on CD4^+^ T cells was examined by flowcytometery. Bar diagrams illustrating CD4 T cells of lungs co-expressing (**d**) IFN-γ^+^TNF-α^+^ and (**e**) IL-17A^+^IFN-γ^+^. Data are representative of 2–3 experiments (n = 3 mice/group) and depicted as means ± SEM. D-L91: mice treated with combinatorial therapy of INH + RFP in drinking water along with L91. ^*^P ≤ 0.05, ^**^P ≤ 0.005, ^***^P ≤ 0.0005, ns = none significant. D-L91: mice administered with drugs in association with L91.

The *Mtb* infected mice were treated with drug and L91 (D-L91), as described in the materials and methods (Fig. 1). In our therapeutic animal model, we sacrificed animals after 20 wk of *Mtb* infection and examined if L91 can rescue CD4 T cells from undergoing exhaustion. Lung resident CD4 T cells from D-L91 inoculated animals were evaluated for the *ex vivo* expression of exhaustion marker PD-1. Noteworthy, CD4 T cells of animals from D-L91 group showed significant (*P* ≤ 0.005) down-regulation in the expression of PD-1, as compared to drugs administered mice (Fig. 2a). These results signify that L91 immunization efficiently restricts CD4 T cells from undergoing exhaustion during long-term exposure of *Mtb* antigens and may be generating qualitative CD4 T cells response. Further, to evaluate the nature of L91 induced immune response, single cell suspensions from spleen and lungs were prepared from treated mice after 20 wk of *Mtb* infection and *in vitro* stimulation with L91, Pam2Cys and medium alone. IFN-γ is a key cytokine that plays an important role to combat *Mtb* by eliciting autophagy and apoptosis upon *Mtb* infection^12^. The secretion of IFN-γ by CD4 T cells is associated with Th1 cells. Recently, the role of IL-17A has been reported in protection by chemokines mediated migration of innate cells and memory Th1 cells^13^,^14^. Isoniazid (INH) and Rifampicin (RIF) are known to suppress the immunity by inhibiting the production of IL-12 and IFN-γ^15^. IL-12 and IFN-γ differentiate the naïve CD4 T cells to Th1 cells^11^,^16^. Consequently, we checked the secretion of IFN-γ and IL-17A by incubating cells isolated from lungs and spleen of D-L91 inoculated mice with L91. We noted substantially greater level of IFN-γ (lungs, spleen: *P* ≤ 0.0005) and IL-17A (lungs, spleen: *P* ≤ 0.0005) with D-L91 compared to drugs administered animals (Fig. 2b,c and Fig. S1a,b). The results of D-L91 were comparable with L91 immunized mice. Thus suggesting that L91 efficacy to elicit immune response was not impaired by drugs.

### L91 exhibited therapeutic efficacy by generating polyfunctional Th1 cells and Th17 cells

The polyfunctional CD4 T cells are considered functionally and qualitatively better than their counter parts secreting single cytokine^17^. To examine L91 induced polyfunctional CD4 T cells, cultures were set as described in materials and methods. Strikingly, D-L91 (lungs: *P* ≤ 0.05, spleen: *P* ≤ 0.05) administered mice generated significantly better L91 specific IFN-γ^+^TNF-α^+^ polyfunctional Th1 cells, as compared to drugs alone (Fig. 2d and Fig. S1c). Similarly, we found remarkable expansion of IL-17A^+^IFN-γ^+^ polyfunctional Th17 cells (lungs: *P* ≤ 0.05, spleen: *P* ≤ 0.005) of D-L91 administered group (Fig. 2e and Fig. S1d).

### Drugs do not interfere in the L91 engendering of enduring memory CD4 T cells in *Mtb* infected animals

**Figure 3:**
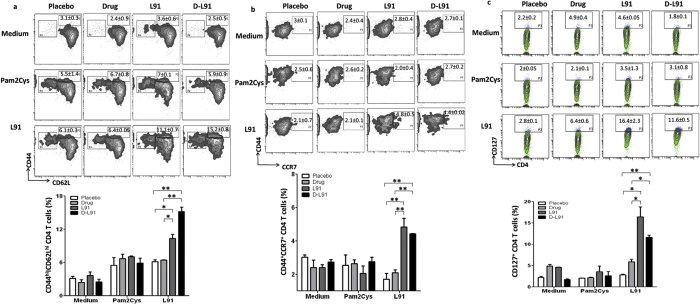
*Mtb* infected mice treated with D-L91 successfully generates enduring memory CD4^+^ T cells. The animals on D-L91 therapy were sacrificed at 20 wk of pi and cells were isolated from spleen and cultured with L91, Pam2Cys and medium. CD4 gated T cells were checked for the expression of memory markers CD62L, CD44, CCR7 and CD127 by flowcytometry. The results depicted as contour plots and bar diagram are the percent population of (**a**) CD62L^hi^CD44^hi^; (**b**) CD44^hi^CCR7^hi^; (**c**) CD127^hi^ CD4 T cells of spleen. The data represented as means ± SEM are of 2–3 experiments (n = 3 mice/group). ^*^P ≤ .05, ^**^P ≤ 0.005.

We have shown earlier that L91 vaccination evokes enduring memory T cells response^11^. The treatment with INH induces apoptosis of CD4 T cells and down-regulates the expression of activation marker CD44 and therefore suppresses the generation of memory T cells^15^. Consequently, it was of concern whether anti-TB drugs used as adjunct therapy may interfere with the L91 property of generating long-lasting memory T cells. Splenic and lung cells of D-L91 group showed considerable increase in the central memory CD4 T cells phenotype CD44^hi^CD62L^hi^ (Fig. 3a and Fig. S2a,b). CD62L^hi^CD44^hi^ are well-established markers for the central memory CD4 T cells responsible for imparting long-lasting protection^11^,^18^,^19^.

CCR7 is also a well-established memory marker of CD4 T cells that is responsible for their migration to the secondary lymphoid organs^20^. Hence, we further confirmed our results of the memory generation and observed remarkable expansion of CCR7^hi^CD44^hi^ exhibiting memory CD4 T cells isolated from the spleen and lungs of D-L91 group compared to drugs alone (Fig. 3b and Fig. S2c,d). It has been well documented that CD127 is essential for the persistence of memory CD4 T cells^21^. Similarly, we observed significant increase in the expression of CD127 on CD4 T cells upon *in vitro* stimulation with L91 in the D-L91 treated group (Fig. 3c and Fig. S2e,f). Thus, signifying that L91 overcomes the drug induced immune suppression. No change was observed in the cells cultured with Pam2Cys or medium alone. Recently, our group has shown that TLR-2 signaling rescues Th1 cells during chronic *Mtb* infection^22^. L91 is comprised of TLR-2 agonist Pam2Cys and CD4 T cell epitope.

### Adjunct therapy of anti-TB drugs with L91 efficiently decreases *Mtb* burden

**Figure 4:**
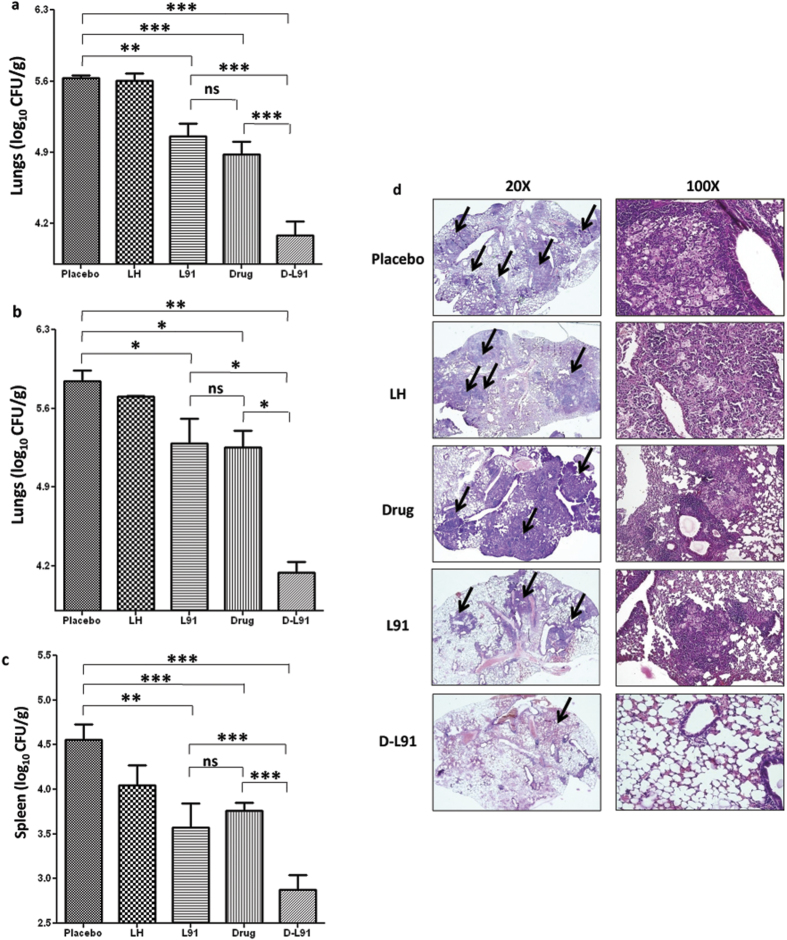
D-L91 substantially decreases bacterial burden in *Mtb* infected animals. The bar diagram indicates pulmonary bacterial burden of D-L91 treated animals that were sacrificed on (**a**) 20 wk and (**b**) 32 wk of pi; (**c**) splenic CFUs at 20 wk of pi; (**d**) histopathology sections by H&E staining (magnification: 20× and 100×) were performed. The arrows indicate granulomas or tubercle and asterisks denote level of significance. (n = 3 mice/group). The data depicted as means ± SEM. are log_10_ CFU/g of tissue. ^*^P ≤ 0.05, ^**^P ≤ 0.005, ^***^P ≤ 0.0005, ns = none significant. PBS: mice administered with PBS, LH: lipidated hemagglutinin peptide, Drug: mice orally fed with INH + RIF in drinking water, D-L91: mice administered with INH + RIF and sc immunized with L91.

To evaluate protective efficacy of combinatorial therapy of drug and L91, *Mtb* infected mice were treated with anti-TB drugs in combination with L91. Four wk of *Mtb* post-infection (pi), mice were orally fed with drugs (INH: 50 mg/L, RIF: 50 mg/L) in drinking water from 4 wk to 8 wk. Animals were sacrificed and bacterial burden in the lungs and spleen were enumerated after 20 wk and 32 wk of pi (Fig. 1). We observed that *Mtb* infected animals treated with INH + RIF in combination with L91 (D-L91) showed a considerable decrease in the bacterial burden in the lungs at 20 wk of pi, as compared to control drugs INH + RIF (*P* ≤ 0.0005) or L91 alone (*P* ≤ 0.0005) (Fig. 4a). We also noted significant reduction in the bacterial load at 32 wk pi, as compared to drugs INH + RIF (*P* ≤ 0.05) or L91 (*P* ≤ 0.05) treated animals (Fig. 4b). Further, we validated that the protection was *Mtb* specific by observing no change in the mice immunized with lipidated peptide of haemaglutinin virus (LH), a non-*Mtb* peptide (Fig. 4a,b). Noteworthy, D-L91 efficiently restricted the dissemination of *Mtb,* as evidenced by considerable (*P* ≤ 0.0005) decline in the CFUs in the spleen at 20 wk of pi (Fig. 4c).

In another set of experiment, *Mtb* infected mice were orally administered twice with a regulated dose of INH (25 mg/kg body wt) at 4 wk and 6 wk of pi (Fig. S3a), as compared to recommended daily administration of same dose^23^. The bacterial burden by CFU in the lungs and spleen of treated animals were enumerated at 20 wk of pi. It was observed that treatment with D-L91 significantly restricted the bacterial burden in the lungs (*P* ≤ 0.05) and spleen (*P* ≤ 0.05), as compared to drug alone (Fig. S3b,c). Thus indicating that regulated delivery of two doses of adjunct therapy of INH with L91 was substantially better than the drug alone in reducing the *Mtb* load.

Further, we validated our CFU data by histopathological analysis of lungs of the infected mice. Animals that received D-L91 showed very small granulomas, leaving most of the normal architecture of lungs. Further, animals from control groups revealed more and larger size of granulomas with macrophages and lymphocytes infiltration and showed irregular morphology of larger expanding areas of consolidation (Fig. 4d).

### L91 stimulates PBMCs of TB patients on drug treatment

**Figure 5:**
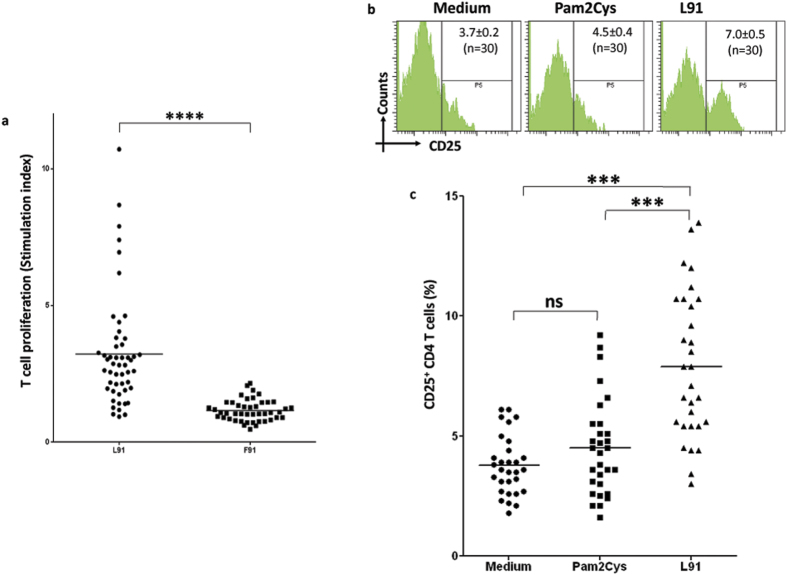
L91 stimulation activates CD4 T cells of TB patients on drug therapy. PBMCs of TB patients were stimulated with L91 and control cultures with F91, Pam2Cys or medium for 96 h. The cells were analyzed for the (**a**) proliferation and expressed as stimulation index; (SI: proliferation in the presence of peptides/proliferation in the absence of peptides). Each dot corresponds to one patient (n = 53). (**b**,**c**) The expression of the activation marker CD25 on CD4 T cells was examined by flowcytometery and shown as (**b**) flowcytometry histograms and (**c**) scatter dot plots, illustrating the percent population CD25 expressing CD4 T cells. Each dot corresponds to one patient (n = 30). F91: non lipidated peptide of 16 kDa antigen of *Mtb*. The data depicted as means ± SEM. ^***^P ≤ 0.0005, ^****^P ≤ 0.0005, ns = none significant.

Next we were curious to monitor the efficacy of L91 on the PBMCs of TB patients on drug therapy. Hence, PBMCs of TB patients who were on Direct Observed Treatment (DOTs) were stimulated *in vitro* with L91. We observed that L91 induced robust proliferation (*P* ≤ 0.0005) and activation (*P* ≤ 0.0005) of CD4 T cells, as revealed by ^3^H-thymidine incorporation assay and CD25 expression, respectively (Fig. 5a–c). No discernible change was seen in the control PBMCs stimulated with un-lipidated peptide (F91) or Pam2Cys. We could not notice any change in the Tregs population with L91 in the cells obtained from PPD^+^ healthy volunteers (Fig. S4a,b). As noted in the case of mice treated with drugs, L91 successfully retained its efficiency to stimulate human CD4 T cells of the TB patients on drug therapy. Therefore, drug regime will not have any influence on the efficiency of L91.

### Treatment of PBMCs of TB patients with L91 augments Th1 and Th17 immunity

**Figure 6:**
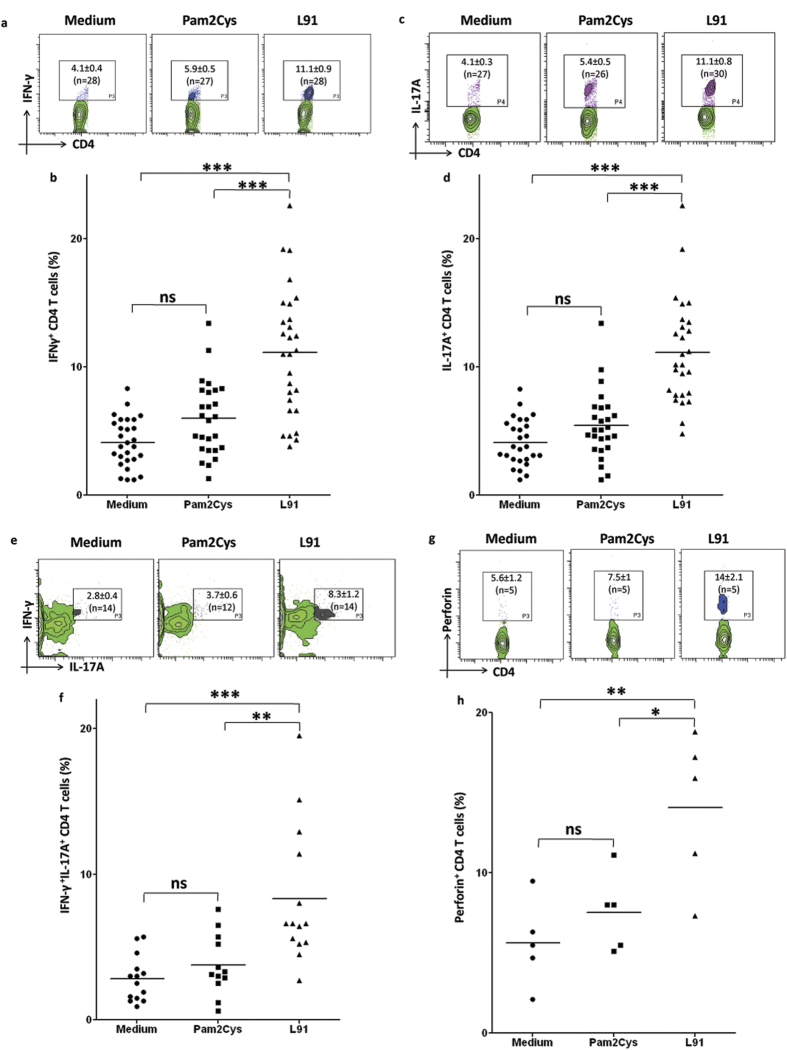
Drug treatment of TB patients does not hamper the efficacy of L91 in the activation of the generation of Th1 and Th17 immunity and memory CD4 T cells. PBMCs of TB patients were cultured with L91 and controls with Pam2Cys and medium for 96 h. Later, CD4^+^ T cells were analyzed for the expression of IFN-γ, IL-17A and perforin. The contour plots of flowcytometery and scatter dot plots represent the percent population of (**a**,**b**) IFN-γ (n = 28), (**c**,**d**) IL-17A (n = 27), (**e**,**f**) co-expression of IFN-γ and IL-17A (n = 14), (**g**,**h**) perforin (n = 5) expressing CD4 T cells. Data depicted as contour and dot plot are percent population of cells. Each dot corresponds to one patient. The data depicted as means ± SEM. ^*^P ≤ 0.05, ^**^P ≤ 0.005, ^***^P ≤ 0.0005, ns = none significant.

In consistency of animal data, we observed that L91 activated Th1 cells and Th17 cells using PBMCs of TB patients on drug therapy, as evidenced by significant expansion in the percent of IFN-γ^+^ (*P* ≤ 0.0005) and IL-17A^+^ (*P* ≤ 0.0005) CD4 T cells (Fig. 6a–d). Further, we also detected remarkable (*P* ≤ 0.005) expansion of IFN-γ^+^ and IL-17A^+^ co-expressing polyfunctional Th17 cells (Fig. 6e,f). Like CTLs, the perforin mediated target-lysis of Th1 cells has been well documented in viral and bacterial diseases^24^,^25^,^26^. Interestingly, we identified high (*P* ≤ 0.05) expression of perforin in the CD4 T cells stimulated with L91 (Fig. 6g–h). These results imply that L91 can efficiently bolster host immunity to protect from *Mtb* infection.

### L91 stimulation of PBMCs of TB patient generated enduring memory CD4 T cells response

**Figure 7:**
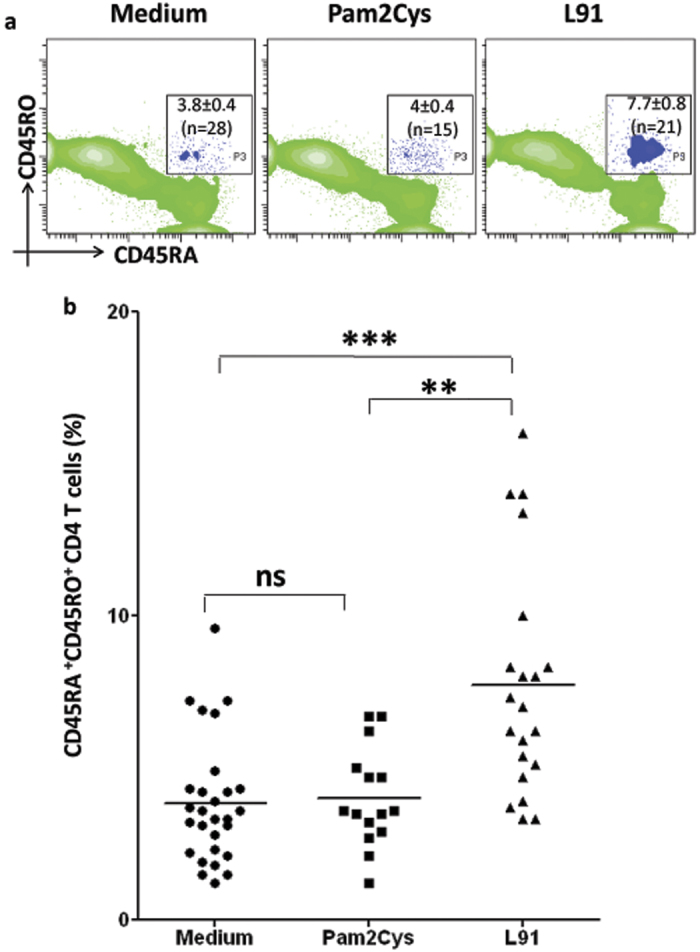
L91 engender CD4 T cell memory obtained from TB patients. PBMCs were isolated from blood obtained from TB patients on drug treatment and *in vitro* incubated with L91, Pam2Cys and medium for 96 h. Later, CD4 gated cells were analyzed for co-expression of CD45RA and CD45RO by flowcytometery. The results (means ± SEM) are depicted as (**a**) contour plots and (**b**) scatter dot plot and correspond to percent population of CD45RA^hi^CD45RO^hi^ CD4 T cells. Each dot corresponds to one patient (n = 28). ^**^P ≤ 0.005, ^***^P ≤ 0.0005, ns = none significant.

The generation of long-term memory T cell response is vital to protect from subsequent infection throughout life-span. Naïve (CD45RA^hi^CD45RO^lo^) human CD4 T cells acquire effector memory (CD45RA^lo^CD45RO^hi^) phenotype upon priming with antigen *via* memory precursor subset co-expressing both CD45RA and CD45RO^27^,^28^. Hence, we thought it would be wise to monitor the generation of such memory precursors after L91 stimulation. Interestingly, employing TB patients PBMCs, we found significant (*P* ≤ 0.005) expansion in the pool of CD45RA^hi^CD45RO^hi^ central memory CD4 T cells. No change was observed in the control cultures incubated with Pam2Cys or medium (Fig. 7a,b). These results suggest that L91 can successfully induce the generation of memory T cells in the patients on drug therapy. Therefore the results illustrate the therapeutic potential of L91 along with promoting the generation of memory CD4 T cells, which are responsible for long-term protection against *Mtb*.

## Discussion

Anti-TB drugs are associated with adverse side-effects like hepatitis, vasculitis, lupus like syndrome, etc^6^,^29^,^30^. Further, in randomized clinical trials severe side-effects of ototoxicity, hepatotoxicity, neuropsychiatric manifestation and hyperuricemia are also reported in hospitalized TB patients undergoing drug treatment^31^. The risk of developing such damaging effects may be more serious with lengthy and high dose of drug regimen. Further, it is also associated with drug induced immune suppression and emergence of drug resistant strains of *Mtb*^32^. Globally, 480,000 MDR TB cases appear annually, which suggest the lack of effective therapy^33^. Recently, XDR and TDR cases are also reported, which pose major challenge for the development of rapid and effective therapies^34^.

L91 is a chimera of promiscuous CD4 T cell epitope of 16 kDa antigen of *Mtb* conjugated to Pam2Cys, a TLR-2 agonist and elicits both innate and adaptive immunity. Pam2Cys of L91 stimulates antigen presenting cells through TLR-2, whereas promiscuous peptide activates enduring memory Th1 cells and Th17 cells^11^. Pam2Cys is also known to rescue Th1 cells from exhaustion^22^. Th1 cells and Th17 cells are considered essential for protection against TB^14^,^35^. L91 protects mice and Guinea pigs from *Mtb* by significantly reducing the bacterial burden in the lungs and spleen. Therefore, we conjecture that this approach of modulating the immune system through L91 and co-administering anti-TB drugs may be quite advantageous in helping to reduce the dose and duration of anti-TB drugs. Many recombinant vaccines like HBHA-hIL12, H56 and DNA expressing Ag85A, Ag85B, ESAT-6 and hsp75 have shown promising results in combination with anti-TB drugs in restricting the bacterial burden in *Mtb* infected animals^32^,^36^,^37^. Therefore, we thought that it would be a judicious idea to explore the therapeutic potential of L91 in conjunction with anti-TB drugs (D-L91) to check the decline in the bacterial burden in the mice infected with *Mtb*. Consequently, we treated mice with RIF and INH along with L91. Following exciting findings have emerged from this study using D-L91 adjunct therapy: i) significant decline in *Mtb* burden and reduction in the number and size of granulomas in the lungs and spleen; ii) considerable protection with two doses of drug and L91; iii) substantial improvement in the pool of polyfunctional Th1 cells and Th17 cells; iv) remarkable decrease in the frequency of CD4 T cells expressing PD-1 marker; v) noteworthy increase in the presence of enduring memory T cells; v) the augmentation of the immune response observed in mice was successfully replicated in the T cells of TB patients on drug therapy.

*Mtb* infected mice treated with D-L91 conferred better protection than drug alone, as authenticated by significant diminution in the bacterial burden in the lungs and spleen. It is worth to mention that we administered suboptimal doses of both INH and RIF. The histopathological analysis also supports the protection data as revealed by regular architecture of lungs. The L91 induced protection was observed even after 32 wk of pi; signifying the establishment of long-term protection. The mechanism involved is due to substantial increase in the immunity, as suggested by improved level of polyfunctional Th1 cells and Th17 cells and enduring memory CD4 T cell response. These results also suggest that combinatorial therapy with L91and anti-TB drugs may offer valuable criteria to protect even at the late stage of chronic infection. Polyfunctional CD4 T cells with concomitant production of both IFN-γ and TNF-α or IFN-γ and IL-17A have been reported to control *Mtb* infection more proficiently than their single cytokine releasing counterparts^11^,^13^. We observed increased levels of IFN-γ along with TNF-α. It has been reported that IFN-γ and TNF-α are considered as potent inducer of autophagy and apoptosis and responsible for killing of *Mtb* infected macrophages^38^,^39^. Th17 cells secreting IL-17 along with IFN-γ are considered as non-pathogenic subset of Th17 cells and protect by recruiting the cells of innate and adaptive immunity and without provoking any deleterious inflammatory response^13^,^14^,^40^,^41^,^42^. Intriguingly, expansion of polyfunctional Th1 cells and Th17 cells and sustenance of memory response in D-L91 administered mice suggest that L91 induced immune response is not suppressed by INH and RIF.

It has been reported that TB patients on drug treatment show downregulation in the level of protective Th1 and Th17 immunity^43^,^44^. Consequently, we also extended our observation to TB patients on DOTs therapy. Since, it may be interesting to monitor whether L91 efficacy in activating T cells is compromised in TB patients on drug therapy. Excitingly, mouse data was successfully replicated with the PBMCs of TB patients. We found that L91 induced significant proliferation and activation of CD4 T cells. Further, we observed enhancement in the levels of Th1 cells and Th17 cells, as evidenced by the secretion of IFN-γ and IL-17A upon *in vitro* stimulation with L91. Furthermore, we noted the generation of IFN-γ and IL-17A secreting polyfunctional Th17 cells in response to L91. This subtype of Th17 cells play an important role in accelerating the recruitment of Th1 cells at the site of infection through induction of CXCR3 and its ligands CXCL9/CXCL10/CXCL11^13^,^14^.

Isoniazid is known to kill replicating bacteria by inhibiting the synthesis of mycolic acid and rifampicin restricts the bacterial proliferation by inhibiting bacterial RNA polymerase^45^,^46^,^47^,^48^. The added advantage of D-L91 therapy is that the drug will kill the proliferating *Mtb*, whereas L91 will not only provoke host immunity to kill replicating but as well eliminate the intracellular hidden bacterium. Finally, L91 induced immunological memory will prevent the host from subsequent encounter with *Mtb*. In future, these findings may have immense clinical implication for treating TB patients by considerably minimizing the dose and duration of current drug regime.

## Methods

### Ethical Statement

All the animal related experiments were approved by the Institutional Animal Ethics Committee of the CSIR-IMTECH, for project license no IAEC 11/21 and experiments were carried out in accordance with the National Regulatory Guidelines issued by the Committee for the Purpose of Supervision of Experiments on Animals (No. 55/1999/CPCSEA), Ministry of Environment and Forest, Government of India. All the human blood related work was strictly conducted in accordance with the ethical guidelines for biomedical research on human subjects by Central Ethics Committee on Human Research (CECHR), ICMR-2000 and those as contained in ‘Declaration of Helsinki’. All the donors provided informed consent in writing. No child was involved in this study. All the experimental protocols were approved by Institutional Animal Ethics Committee of the CSIR-IMTECH for project license no IAEC 11/21 and Institutional ethics committee of the Government Medical College and Hospital, Chandigarh for project license GMC/TA-I (19D) 2010/06776.

### Immunization and infection

Female BALB/c mice (6–8 wk) were procured from Experimental Animal Facility, CSIR-Institute of Microbial Technology, Chandigarh, India. Mice were aerosol challenged with *Mtb* (100 CFU/mouse). Later, mice were immunized twice subcutaneously (sc) at the base of the tail at 4 wk and 6 wk post-infection (pi) with 20 nmol and 10 nmol of L91, respectively. Drug treated groups were orally administered isoniazid (INH, 50 mg/L) and rifampicin (RIF, 50 mg/L) in drinking water from 4 wk to 8 wk of pi. Control groups were immunized with placebo (PBS), LH and INH + RIF. Bacterial burden in the lungs and spleen were enumerated through CFUs at 20 wk and 32 wk of pi.

### Lipidated peptides used in the study

Lipidated promiscuous peptide (L91) used in the study of sequence SEFAYGSFVRTVSLPVGADE was the immunodominant epitope of 16 kDa antigen of *Mtb* conjugated with Pam2Cys. It was synthesized, purified and characterized as described elsewhere^49^. The control non-mycobacterial lipidated peptide of sequence ALNNRFQIKGVELKS was from the light chain of influenza virus hemaglutinin.

### Reagents and antibodies

The chemicals and reagents were purchased from Sigma Aldrich (St. Louis, MO). Anti–mouse flurochrome labeled Abs: CD4-PB, CD62L-APC, CD44-PerCP-Cy5.5, CD127-PE, FoxP3-FITC, PD1-PECy7, CCR7-APC, IFN-γ-PECy7, TNFα-PerCPCy5.5, IL-17-PerCPCy5.5 and Abs for ELISA were procured from BD Pharmangin (San Diego, CA) or otherwise mentioned. RPMI-1640 and FBS were purchased from GIBCO (Grand Island, NY). Tissue culture grade plastic-ware was purchased from BD Biosciences (Bedford, MA).

### Culture of spleen and lung lymphocytes

Spleens and LNs obtained from experimental mice were pooled and gently pressed through frosted slides for preparing single cell suspension. The lungs were perfused by chilled PBS and small pieces were prepared and digested with collagenase (2 mg/ml) for 30 min/37 °C. Later, cells were passed through strainer (70 μM). Cell viability was assessed by trypan blue exclusion method. Splenocytes/lung cells (2 × 10^5^/well) were cultured with L91 (1 nmol), Pam2Cys (50 ng/ml) and medium in 96-well U bottom plates for 72 h.

### Stimulation of TB patient PBMCs with L91

The blood of sputum positive pulmonary TB patients on drug therapy for 2–6 months were collected in sterile vacutainers. The blood was diluted with 1X PBS in 1:1 ratio and peripheral blood mononuclear cells (PBMCs) were separated through ficoll-histopaque gradient centrifugation method (400 g/30 min/25 °C). PBMCs were washed 3X with 1X PBS + 2% FCS and *in vitro* cultured with L91 (1 nmol), F91 (1 nmol) or Pam2Cys (50 ng/ml) for 96 h. During culture IL-2 (100U) was added after 24 h. All the human related blood work was approved (ref. 06776/4/2/2010) by ‘Institutional Ethics Committee Government Medical College and Hospital, Chandigarh’ and performed according to the guidelines of ‘Indian Council of Medical Research, Government of India. All adult blood donors provided informed consent in written form.

### Proliferation Assays

The cells (2 × 10^7^) isolated from spleen + LNs and lungs were incubated with carboxyfluoresceinsuccinimidyl ester (CFSE) (2 μM) in PBS for 8 min/37 °C. Excess CFSE was removed by extensive washing with RPMI-FCS-10%. CFSE labeled cells (2 × 10^5^ cells/well) were cultured with L91 (1 nmol) and Pam2Cys (50 ng/ml) and medium for 72 h. The proliferation of CFSE-labeled cells was analyzed by flowcytometry. The proliferations of human T cells were monitored by stimulating human PBMCs with L91 or F91 for 72 h. Later,^3^H-thymidine (1 μCi/well) was added in the cultures. After 16 h, plates were harvested and radioactivity incorporated was measured by liquid scintillation counting.

### Flowcytometry staining

The cells were stimulated with L91 as mentioned in the proliferation assay. For surface staining, cells were incubated with either fluorochrome labeled Abs or biotinylated Abs/streptavidin-fluorochrome conjugates along with their respective isotype matched control Abs. For intracellular cytokine staining, L91 cultured cells were restimulated with phorbol 12-myristate 13-acetate (PMA) (50 ng/ml) and ionomycin (1 μg/ml) for 4 h followed by incubation with brefeldin A (5 mg/ml) for an additional 2 h. Afterward, cells were harvested, transferred to tubes and washed 2X with PBS (1XPBS-FBS-2%), followed by fixation with paraformaldehyde (1X) at 4 °C/30 min. The cells were then perforated with saponin (0.2%) and stained with fluorochrome-labeled Abs and isotype-matched control Abs. Standard protocols of washing/incubation were followed at each stage. Samples were acquired on FACS-Aria III and analyzed using FACS DIVA software (BD Biosciences, San Jose, CA).

### Cytokine estimation

Supernatants from cultures were collected after 72 h and cytokines were estimated by sandwich ELISA as per manufacturer instructions^50^.

### Statistical analysis

The data are mainly presented as means ± SEM. Statistical analysis was performed employing an unpaired Student’s ‘t’ Test.

## Additional Information

**How to cite this article**: Rai, P. K. *et al*. A novel therapeutic strategy of lipidated promiscuous peptide against *Mycobacterium tuberculosis* by eliciting Th1 and Th17 immunity of host. *Sci. Rep.* **6**, 23917; doi: 10.1038/srep23917 (2016).

## Supplementary Material

Supplementary Information
