# Supplementary material for: A novel therapeutic strategy of lipidated promiscuous peptide against Mycobacterium tuberculosis by eliciting Th1 and Th17 immunity of host
Source: Sci Rep. 2016 Apr 7;6:23917. doi: 10.1038/srep23917 (PMC4823727; doi:10.1038/srep23917)
Supplement: Supplementary Information [file srep23917-s1.pdf]

A novel therapeutic strategy of lipidated promiscuous peptide against *Mycobacterium tuberculosis* by eliciting Th1 and Th17 immunity of host

Pradeep K Rai, [£]Sathi Babu Chodisetti, Sajid Nadeem, Sudeep K Maurya, [δ]Uthaman Gowthaman, *Weiguang Zeng, [†]Ashok K Janmeja, *David C Jackson, Javed N Agrewala[#]

CSIR-Institute of Microbial Technology, Chandigarh, India, *Department of Microbiology andImmunology, Peter Doherty Institute for Infection and Immunity, The University of Melbourne, Parkville 3010, Victoria, Australia,[†]Department of Pulmonary Medicine, Government Medical College and Hospital, Chandigarh, India

[£]Current address: Department of Microbiology and Immunology, Pennsylvania State University College of Medicine, Hershey, PA 17033, USA

[δ]Current address: Department of Orthopedics, Yale School of Medicine, New Haven, CT, USA,

Short running title: Tuberculosis therapy by lipidated peptide

Key words: TB patients, Vaccine, promiscuous peptide, Pam2Cys, TLR-2, Isoniazid, Rifampicin, tuberculosis, *Mycobacterium tuberculosis*, Th1 cells, Th17 cells

Correspondence should be addressed to J.N.A (javed@imtech.res.in)

Supplementary Figures

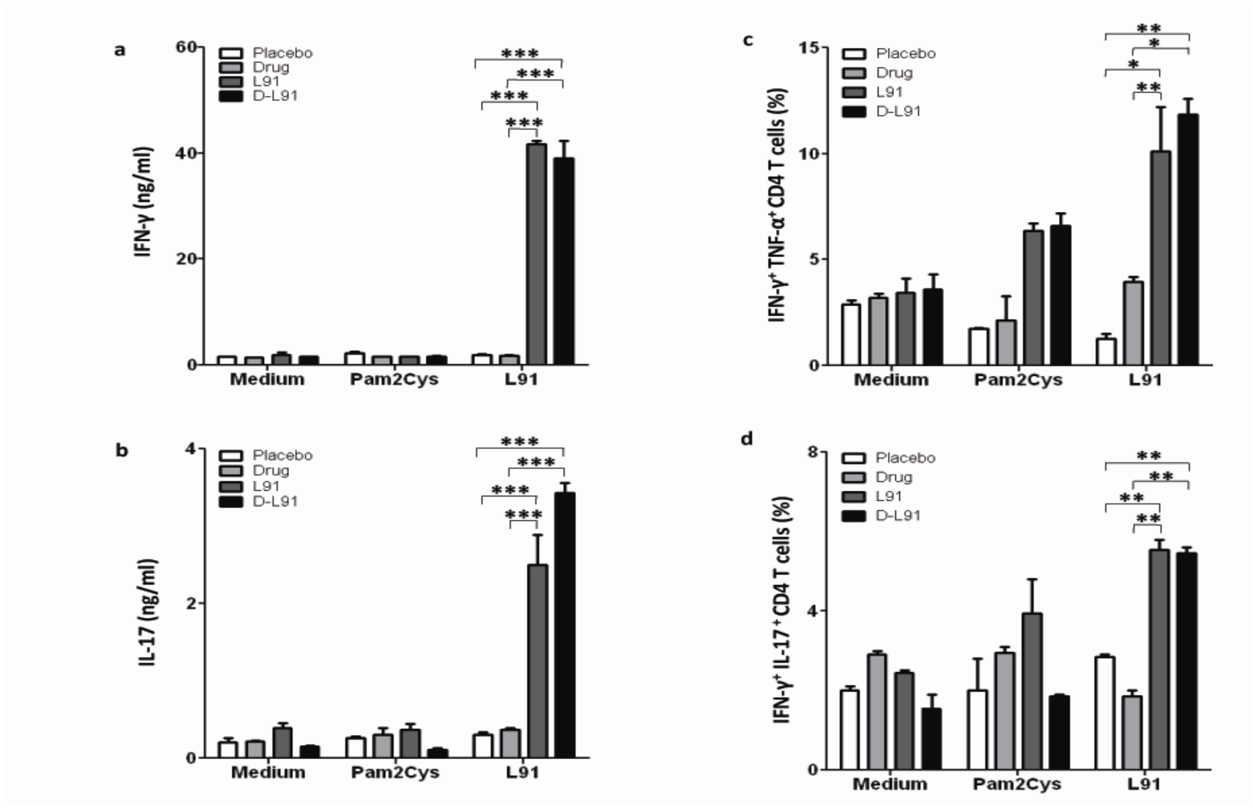

Figure S1. L91 immunization induces robust Th1 and Th17 immune response. At 20 wk of pi, mice were sacrificed and single cell suspension was prepared from the spleen. The cells were *in vitro* challenge with L91 and control cultures with Pam2Cys and medium. The release of IFN-γ and IL-17A were examined in the SNs of *in vitro* stimulated cultures by ELISA. Bar diagrams indicate release of (a) IFN-γ and (b) IL-17A. The co-expression of IFN-γ/TNF-α and IL-17A/IFN-γ were assessed by intracellular staining and analyzed by flowcytometery. Bar diagrams represent the percentage of (c) IFN-γ^{+}TNF-α^{+} and (d) IL-17A^{+}IFN-γ^{+} CD4 T cells. Data shown are means ± SEM and representative of 2-3 independent experiments (n=3 mice/group). *$P \leq 0.05$, ** $P \leq 0.005$, *** $P \leq 0.0005$.

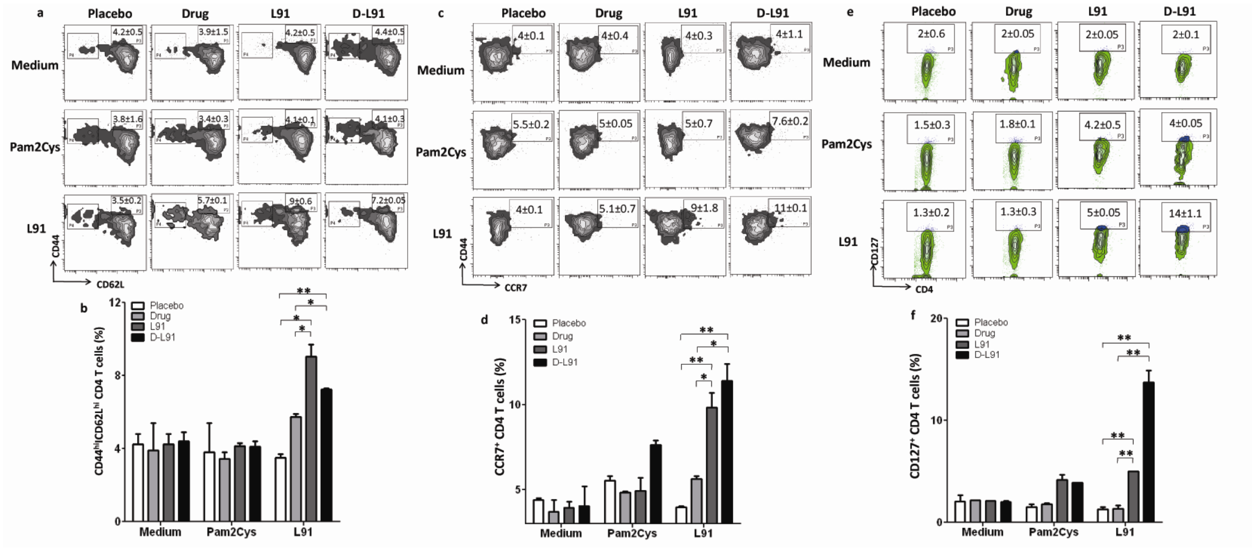

Figure S2. L91 immunization induces memory response in *Mtb* exposed animals. *Mtb* infected animals treated with D-L91 and controls with L91, drug and placebo PBS were assayed for the expression of memory markers CD62L, CD44, CCR7 and CD127 by flowcytometry. Lung cells were *in vitro* stimulated with L91, Pam2Cys and medium. The results portrayed as contour plots and bar diagrams are the percent population of lung CD4 T cells expressing memory phenotype for (a,b) $CD62L^{hi}CD44^{hi}$; (c,d) $CD44^{hi}CCR7^{hi}$; (e,f) $CD127^{hi}$. Data represented as means ± SEM are of 2-3 experiments (n=3 mice/group). *$P \leq 0.05$, **$P \leq 0.005$.

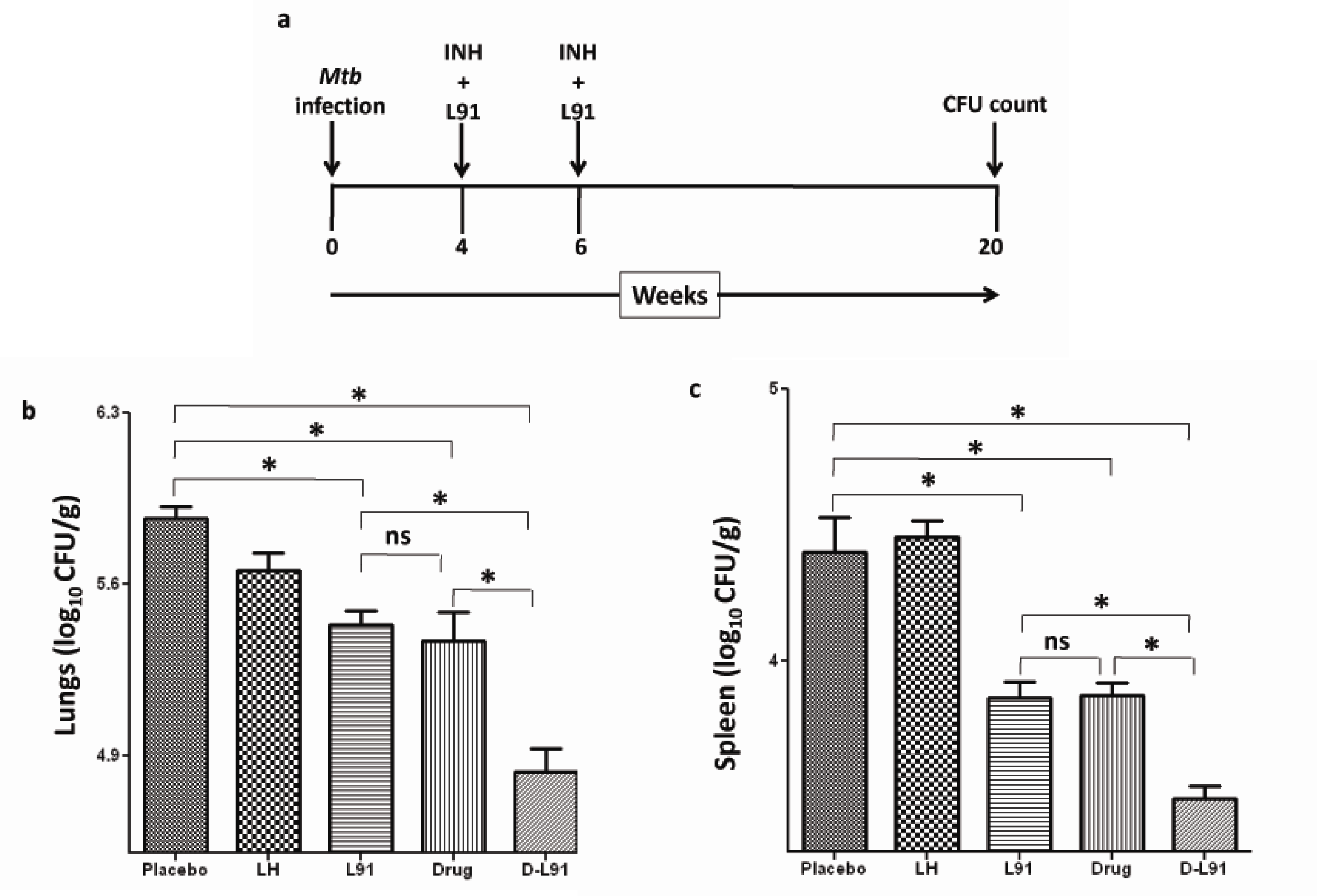

Figure S3. Regulated delivery of two doses of INH and L91 significantly restricts the *Mtb* load. (a) The mice were aerosol infected with ~100 CFU of *Mtb* H37Rv strain and inoculated two doses of INH in combination with L91 at 4 wk and 6 wk of pi. The control groups received PBS, LH, L91 and INH. The bacterial burden in the lungs and spleen was enumerated after 20 wk of pi. The bar diagrams represent CFU of (b) lungs and (c) spleen. The data (mean ± SEM) are represented as log_{10} CFU/g of tissue. *$P \leq 0.05$. PBS: mice administered with PBS, LH: lipidated hemagglutinin peptide, Drug: mice orally administered twice with regulated doses of INH, D-L91: mice administered with INH and L91. *$P \leq 0.05$, ns: non-significant.

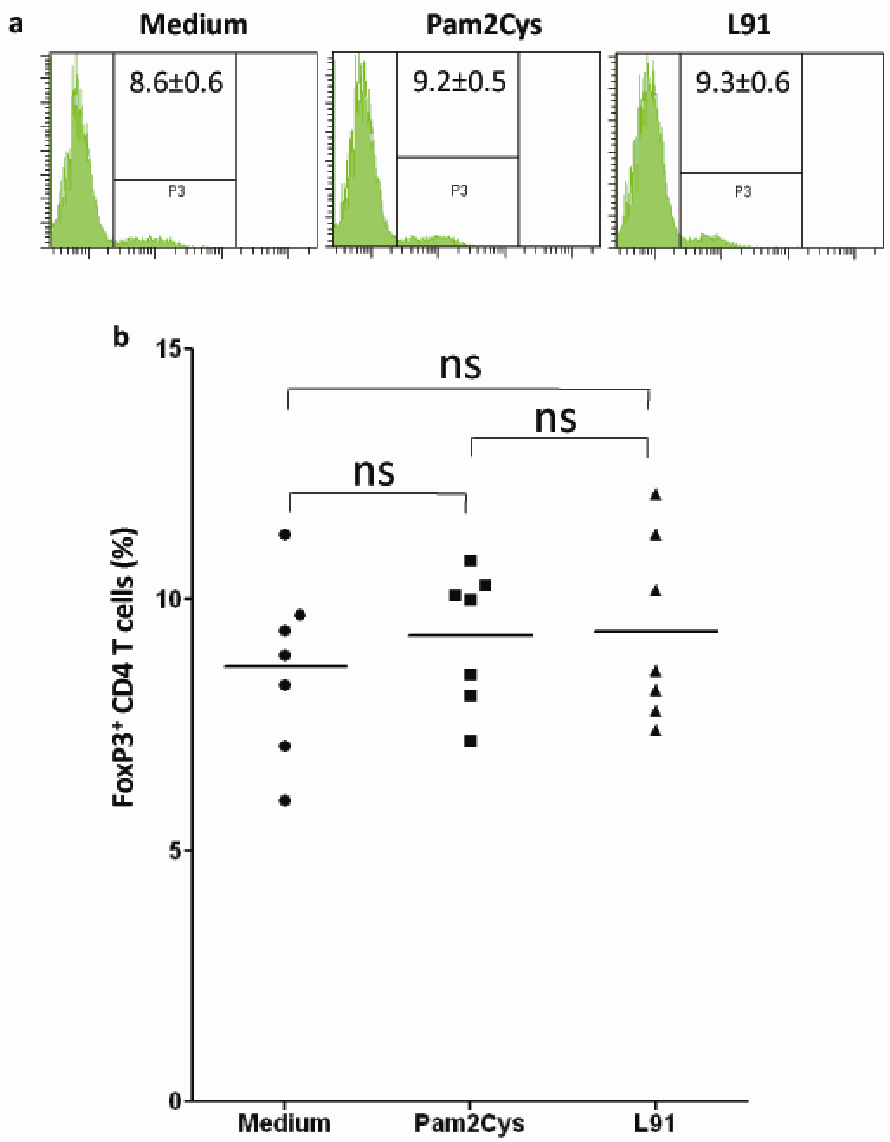

Figure S4. L91 does not induce the generation of Tregs. PBMCs of PPD$^+$ healthy volunteers were stimulated with L91, Pam2Cys and medium alone. After 72h, PBMCs were harvested and expression of FoxP3 was examined on CD4 T cells by flowcytometery. (a) Histogram and (b) scatter dot plot correspond to the percent population of FoxP3 expressing CD4 T cells. Each dot denotes one human subject. The data are expressed as means ± SEM. ns: non-significant.
